# Intracellular Colocalization of Influenza Viral RNA and Rab11A Is Dependent upon Microtubule Filaments

**DOI:** 10.1128/JVI.01179-17

**Published:** 2017-09-12

**Authors:** Eric Nturibi, Amar R. Bhagwat, Stefanie Coburn, Mike M. Myerburg, Seema S. Lakdawala

**Affiliations:** a Department of Microbiology and Molecular Genetics, University of Pittsburgh School of Medicine, Pittsburgh, Pennsylvania, USA; b Division of Pulmonary, Allergy, and Critical Care Medicine, University of Pittsburgh, Pittsburgh, Pennsylvania, USA; St. Jude Children's Research Hospital

**Keywords:** influenza virus, microtubules, Rab11A, endosomes, assembly, RNA, fluorescent *in situ* hybridization, viral RNA

## Abstract

Influenza A virus (IAV) consists of eight viral RNA (vRNA) segments that are replicated in the host cell nucleus and transported to the plasma membrane for packaging into progeny virions. We have previously proposed a model where subcomplexes of vRNA are exported from the nucleus and assembled en route to the plasma membrane. However, the role of host cytoskeletal proteins in the cytoplasmic assembly of IAV vRNA segments remains unknown. Previous studies have suggested that IAV vRNA segments are transported via Rab11A-containing recycling endosomes (RE) and use both microtubules (MT) and actin. Rab11A RE transport primarily along MT; therefore, investigation of the role of MT in vRNA assembly is warranted. We explored the role of MT in vRNA assembly and replication by using multiple IAV strains in various cell types, including primary human airway epithelial cells. We observed that Rab11A localization was altered in the presence of MT-depolymerizing drugs, but growth of IAV in all of the cell types tested was unchanged. Fluorescent *in situ* hybridization was performed to determine the role of MT in the assembly of multiple vRNA segments. Unexpectedly, we found that vRNA-vRNA association in cytoplasmic foci was independent of MT. Given the disparity of localization between Rab11A and vRNA segments in the absence of intact MT filaments, we analyzed the three-dimensional spatial relationship between Rab11A and vRNA in the cytoplasm of infected cells. We found that Rab11A and vRNA colocalization is dependent upon dynamic MT filaments. Taken together, our data suggest that cytoplasmic transport of influenza vRNA may include a Rab11A RE-independent mechanism.

**IMPORTANCE** IAV infections cause a large public health burden through seasonal epidemics and sporadic pandemics. Pandemic IAVs emerge through reassortment of vRNA in animal or human hosts. Elucidation of the mechanism of intracellular dynamics of IAV assembly is necessary to understand reassortment. Our results describing the role of MT in vRNA transport and assembly expand upon previous studies characterizing vRNA assembly. This study is the first to assess the role of MT in influenza virus replication in human bronchial airway epithelial cells. In addition, we present novel data on the role of MT in facilitating the association between distinct vRNA segments. Interestingly, our results suggest that progressive assembly of vRNA segments may be cell type dependent and that vRNA may be transported through the cytoplasm without Rab11A RE in the absence of intact MT. These results enhance our understanding of vRNA assembly and the role of cytoskeletal proteins in that process.

## INTRODUCTION

The influenza A virus (IAV) genome comprises eight negative-sense, single-stranded RNA segments. Each viral RNA (vRNA) segment is associated with viral nucleoproteins (NP) and bound to the virus-encoded heterotrimeric polymerase complex, PB1, PB2, and PA. Synthesis of the IAV vRNA and assembly into viral ribonucleoprotein (vRNP) complexes occur in the nucleus (1). Newly synthesized vRNP segments are transported from the nucleus to the plasma membrane for packaging and budding. All eight vRNP segments are selectively packaged into budding virions (2–5). Evidence suggests that selective vRNP assembly is mediated through direct RNA-RNA interactions between segments (6–8). We and others have previously proposed a mechanism of influenza vRNA assembly where segments are exported from the nucleus as subcomplexes and selective assembly of all eight segments occurs through dynamic fusion and “kissing” events in the cytoplasm en route to the plasma membrane (9, 10).

The host factors mediating vRNP transport from the nucleus to the plasma membrane have been an active area of study, and Rab11A-containing recycling endosomes (RE) have been implicated in vRNP transport (11–14). Rab11 proteins are small GTPases that regulate exocytic processes of the trans-Golgi network and apical transport pathways of RE toward the cell surface (10, 15). Rab11A directs endosomal movement primarily on microtubules (MT) but can also transport along actin filaments via various Rab11A effector proteins (FIPs) associating with MT or actin motor proteins (16, 17). Rab11A interacts with Kif3b, a component of the kinesin II motor protein, through the effector FIP5 protein for the anterograde transport of RE on MT (18). In addition, Rab11A can interact with the effector myosin Vb via FIP2 for transport along actin microfilaments (19). The promiscuous nature of Rab11A RE may be a benefit during vRNP transport. Recent studies have demonstrated a direct interaction between PB2 and Rab11A during a productive viral infection, solidifying the role of Rab11A RE during vRNP cytoplasmic transport (20).

Previously published work examining the role of cytoskeletal proteins during the transport of influenza vRNPs used drugs to alter the structure of MT and actin filaments. Unfortunately, those studies failed to provide a clear picture of the importance of these cytoskeletal filaments during influenza virus assembly. Treatment of infected cells with actin polymerization inhibitors such as cytochalasin D had a modest effect on viral replication (21–24). Similarly, treatment with an MT-depolymerizing agent, nocodazole, a few hours after infection at a high multiplicity of infection (MOI) resulted in only a modest reduction in viral titers during a single-cycle growth curve, suggesting that cytoplasmic vRNP transport does not proceed exclusively along MT (11, 25). A previous study disrupted both MT and actin for a short period of time and found reduced trafficking of vRNP in the cytoplasm when using a virus that produces PB2–split-green fluorescent protein (GFP) (26). However, cells lacking both MT and actin are not viable for long periods of time, which prevents analysis of viral replication in these cells. Importantly, transport of vRNPs from the nucleus is thought to initiate at the MT organizing center (MTOC) with the cellular factor YB-1 and then proceed to the endocytic recycling compartment and Rab11A-containing RE (25, 27, 28). Therefore, the lack of a robust reduction of viral titers in cells treated with MT-depolymerizing agents is surprising, given the clear importance of Rab11A and YB-1 during vRNP transport (13, 29). These studies support the notion that vRNP transport is a complex process that requires further study.

Our previously proposed model of influenza vRNP assembly suggests that Rab11A RE-containing vRNPs undergo dynamic fusion or “kissing” events to facilitate the exchange of vRNPs and produce a complex of all eight vRNP segments (10). To expand upon this model, we hypothesize that transport of Rab11A RE on MT would enable these “kissing” interactions, enhancing the assembly of all eight vRNP segments. Therefore, in this study, we utilized fluorescent *in situ* hybridization (FISH) to investigate the intracellular cytoplasmic vRNP subcomplexes in the presence or absence of MT-depolymerizing drugs in multiple cell types. We provide evidence that multicycle IAV replication is not dependent upon MT in multiple cell culture models. Surprisingly, we observed no dramatic difference in the composition of cytoplasmic foci containing vRNP segments in the presence or absence of intact MT filaments. However, importantly, we found that vRNP and Rab11A association was dependent upon the presence of intact MT filaments, suggesting a potential Rab11A RE-independent mechanism of vRNP cytoplasmic transport in the absence of intact MT filaments.

## RESULTS

### Intracellular distribution of Rab11A and vRNP is disrupted in the absence of MT filaments.

**FIG 1:**
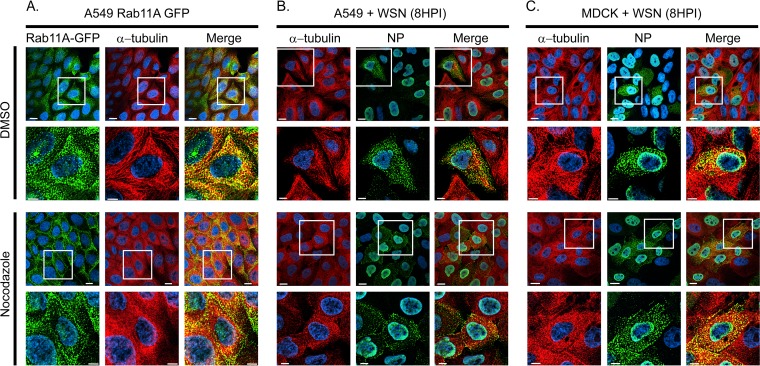
Relocalization of Rab11A and influenza vRNP cytoplasmic distribution with nocodazole treatment. (A) A549 cells stably expressing GFP-tagged Rab11A were treated with DMSO or nocodazole (5 μg/ml) for 4 h prior to fixation. A549 (B) and MDCK (C) cells were infected with WT WSN (MOI of 3) and treated with DMSO or nocodazole (5 μg/ml) at 4 hpi. Cells were stained with anti-alpha-tubulin and anti-NP antibodies at 8 hpi to determine the localization of vRNP in influenza virus-infected cells. Images obtained with a 60× objective are displayed to provide a global perspective of the samples. Zoomed-in areas of the cells (white-outlined boxes) are shown (rows 2 and 4) to display a single representative cell to highlight the MT structure and Rab11A or NP localization. All images are deconvolved, single confocal slices. DAPI (blue) marks the cell nuclei, and the scale bars are 5 μm in all images.

Rab11A RE have been implicated in the cytoplasmic transport of influenza vRNA segments to the plasma membrane for packaging and budding (12–14). To assess the importance of MT and Rab11A RE in the transport of vRNA, we generated A549 cells stably expressing Rab11A tagged with full-length GFP (Rab11A-GFP). These cells were treated with the MT-depolymerizing drug nocodazole to assess whether MT contributes to Rab11A RE localization (Fig. 1A). In line with a previous study (30), we observed that exogenously expressed Rab11A-GFP is concentrated near the nucleus at the MTOC and dispersed throughout the cytoplasm in discrete foci in mock (i.e., dimethyl sulfoxide [DMSO])-treated cells. Treatment of A549 Rab11A-GFP cells with nocodazole resulted in relocalization of the Rab11A-GFP signal from the perinuclear region toward the plasma membrane (Fig. 1A), indicating that the intracellular distribution of Rab11A is dependent upon MT in cells stably expressing tagged Rab11A, similar to a previously published report on endogenous Rab11A (31).

Given the redistribution of Rab11A upon nocodazole treatment, we explored the localization of influenza vRNP in cells treated with nocodazole. A549 and Madin-Darby canine kidney (MDCK) cells were infected with a recombinant WSN influenza virus and treated with nocodazole 4 h later to focus on postentry events. Localization of NP and MT was assessed at 8 h postinfection (hpi) (Fig. 1B and C). We found that localization of vRNPs was altered in the presence of nocodazole, an observation consistent with a previously published report (11). However, the relocalization of NP did not appear to be as extensive as that of Rab11A-GFP in nocodazole-treated cells, since an NP signal was still observed in the cytoplasm while Rab11A-GFP was found predominantly on the plasma membrane and not in the cytoplasmic space (Fig. 1A). These data suggest that the localization of Rab11A and, to a lesser extent, vRNP is dependent upon MT filaments.

### Replication of H1 and H3 influenza viruses in MDCK, A549, and human bronchial epithelial (HBE) cells is independent of MT filaments.

**FIG 2:**
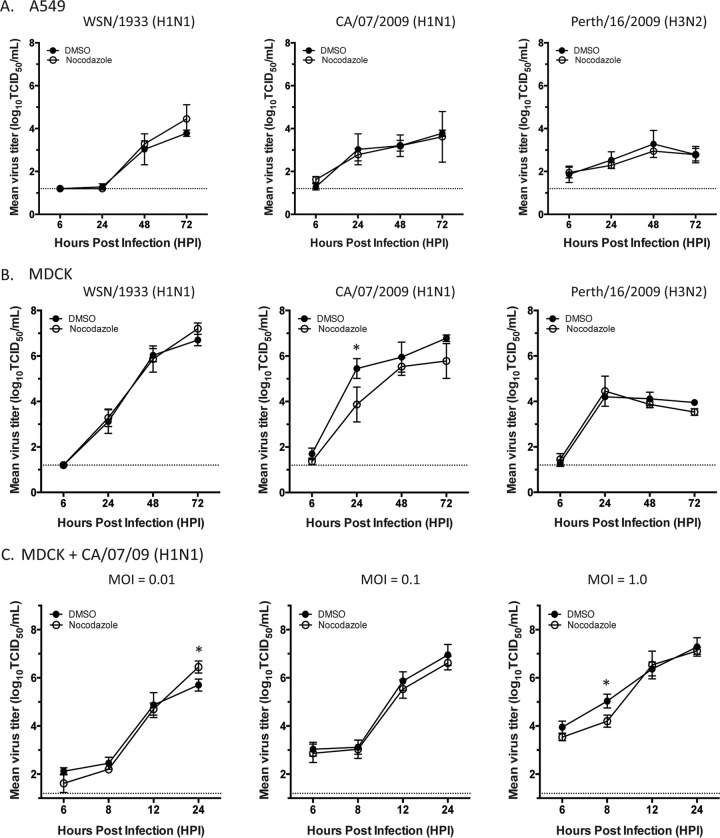
Replication of H1 and H3 viruses in nocodazole-treated cells. MDCK (A) and A549 (B) cells were infected with A/WSN/1933 (H1N1), A/CA/O7/2009 (H1N1), and A/Perth/16/2009 (H3N2) viruses at an MOI of 0.01. Cells were treated with DMSO or nocodazole (5 μg/ml) at 4 hpi, and supernatants were harvested at 6, 24, 48, and 72 hpi for titration on MDCK cells. (C) Single-cycle growth curves of A/CA/O7/2009 (H1N1) in MDCK cells determined at different MOIs as indicated. Cells were treated with DMSO or nocodazole (5 μg/ml) at 4 hpi, and supernatants were harvested at 6, 8, 12, and 24 hpi for titration on MDCK cells. Virus titers were determined by the endpoint method. Values presented are means from three independent experiments, and standard deviations are represented by error bars. A two-way analysis of variance with multiple comparisons was used to define statistically significant differences (marked by asterisks). The dashed line denotes the limit of detection for the titration assay.

Given the redistribution of Rab11A and NP localization in cells treated with nocodazole, we proceeded to investigate if viral replication in different biologically relevant cell types is dependent upon MT. Previous studies have shown a negligible decrease in virus production in nocodazole-treated MDCK cells compared to that in untreated cells by using a high MOI and a single-cycle growth curve (25). We expanded upon these observations by investigating the replication of H1 and H3 IAV in multiple cell types by using a low MOI and a multicycle growth curve to enhance any impact of nocodazole treatment on influenza virus replication (Fig. 2). First, we measured the replication kinetics of human seasonal H1N1 and H3N2 strains in common cell culture cells, MDCK and A549 cells. Cells were infected with a low MOI of strain A/WSN/1933 (H1N1), A/California/07/2009 (H1N1), or A/Perth/16/2009 (H3N2) and then treated with either DMSO or nocodazole at 4 hpi. The replication titers of all of these influenza virus strains were higher in MDCK cells than in A549 cells at all of the time points tested. Consistent with previously published results (25), we found that all of the virus strains tested replicated to similar titers in DMSO- and nocodazole-treated MDCK and A549 cells, except for A/California/07/2009 (H1N1) in MDCK cells at 24 hpi, where the viral titers in DMSO- and nocodazole-treated cells were significantly different (Fig. 2B). To determine if this difference was due to altered vRNP transport or a consequence of the subsequent infections, we determined a series of single-cycle growth curves for A/California/07/2009 (H1N1) in MDCK cells at various MOIs (Fig. 2C). A modest, yet significant, decrease was observed at 24 hpi for an MOI of 0.01, as expected, but not earlier, suggesting that nocodazole does not have an impact on egress events but only affects entry events. Interestingly, no defect was seen at an MOI of 0.1 at any of the time points tested but a difference was observed at 8 hpi in cells infected at an MOI of 1 (Fig. 2C). However, this defect is similar to that observed previously at 8 hpi when using a high MOI (11). Our data suggest that nocodazole has a more pronounced effect on A/California/07/2009 (H1N1) entry into MDCK cells than on its entry into A549 cells and on the WSN and H3N2 virus strains. On the basis of Fig. 2, the impact of nocodazole on virus infection is strain dependent, cell type dependent, and MOI dependent, demonstrating the need to study these factors with a variety of viruses and cell culture systems.

**FIG 3:**
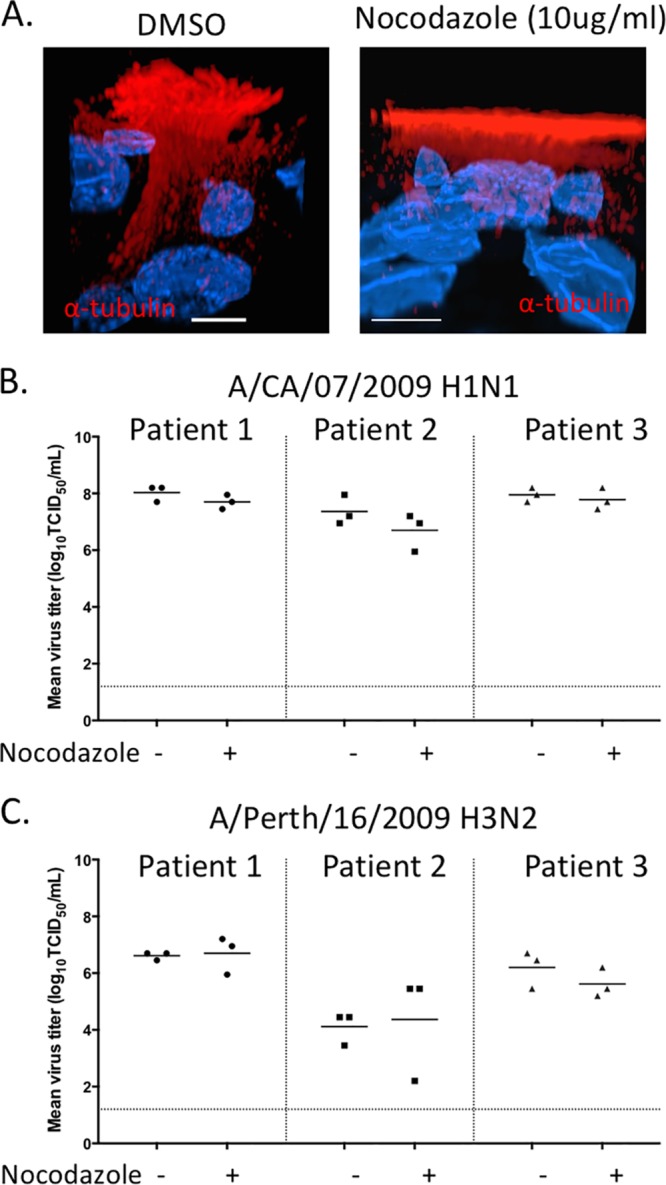
Growth of influenza viruses in HBE cells treated with nocodazole. (A) Uninfected primary HBE cells grown at an air-liquid interface were treated with DMSO or nocodazole (10 μg/ml) on the apical and basal surfaces for 24 h. Fixed transwells were stained with anti-α-tubulin antibody. Intracellular MT and formation of cilia were determined by z-stack acquisition on a confocal microscope. The front view of the cells is displayed by using a clipping plane in the Imaris software. Scale bars are 5 μm. Single representative cells chosen from the entire coverslip are displayed for clarity. HBE cells were infected with A/CA/07/2009 (H1N1) (B) and A/Perth/16/2009 (H3N2) (C) viruses at 10^3^ TCID_50_ per well. At 24 hpi, cells were treated with DMSO(−) or nocodazole (10 μg/ml) on the apical and basal surfaces. Medium containing DMSO and nocodazole was maintained on the apical and basal surfaces for the duration of the experiment. The apical supernatant was collected at 48 hpi for viral titration. This experiment was conducted in triplicate, displayed as each data point, with three different patient cell lines as shown.

Previously published studies on the role of MT filaments on influenza virus replication have all used transformed cell lines, which may not always mimic airway epithelial cells of the respiratory tract naturally infected with influenza viruses. Therefore, we assessed whether MT are critical for the replication of human seasonal viruses A/Perth/16/2009 (H3N2) and A/California/07/2009 (H1N1) in primary HBE cells grown at an air-liquid interface (Fig. 3). HBE cells grown at an air-liquid interface differentiate into a three-dimensional (3D) culture representative of the airway anatomy (32) and therefore represent the most biologically relevant cell types available for *in vitro* studies. Replication kinetics of IAV in HBE cells treated with nocodazole required that medium containing nocodazole be maintained at the apical surface of the HBE cells for 24 h. Treatment of HBE cells with nocodazole for 24 h resulted in a severe disruption of MT, as observed by staining of cells for α-tubulin and observing a distinct change in the morphology of the cilia (Fig. 3A). Interestingly, the replication of H1N1 and H3N2 influenza viruses was not significantly different in nocodazole- and DMSO-treated HBE cells (Fig. 3B and C). These data confirm that MT are not essential for efficient replication of influenza viruses in airway epithelial cells.

### Acetylation of MT is lost during nocodazole treatment.

**FIG 4:**
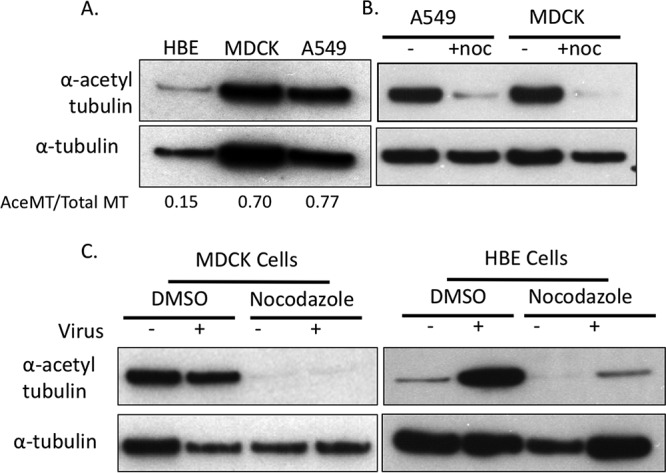
Nocodazole treatment diminishes intracellular acetylation of MT. (A) Western blot analysis of total cell lysates prepared from HBE, MDCK, and A549 cells. Relative levels of acetylated MT were determined with ImageJ and compared to endogenous α-tubulin levels. (B) A549 and MDCK cells were treated with DMSO(−) or nocodazole (5 μg/ml) for 4 h. Acetylated α-tubulin and α-tubulin were detected via Western blotting, which showed that nocodazole treatment decreased the presence of acetylated MT. (C) MDCK cells infected with WSN/1933 (H1N1) were treated with nocodazole (5 μg/ml) and fixed at 24 hpi. HBE cells infected with A/CA/07/2009 (H1N1) were treated with nocodazole (10 μg/ml) and fixed at 48 hpi. Total cell lysates were prepared from the cells, and acetylated α-tubulin and α-tubulin were detected by Western blotting. Nocodazole treatment resulted in a decrease in acetylated tubulin regardless of the cell type and the presence of virus.

Posttranslational modifications of MT, such as acetylation, have been suggested to alter sensitivity to depolymerizing drugs such as nocodazole (33, 34). Therefore, it is possible that Rab11A RE-containing vRNP segments are transported along acetylated MT. The role of acetylated MT in influenza virus infection has been previously studied, and IAV infection was shown to increase the levels of acetylated MT (Fig. 4C) (35). To further investigate the role of acetylated MT in vRNP transport, we measured the levels of acetylated MT in all of the cell types we have studied so far. We observed that different cell types have differing amounts of MT acetylation (Fig. 4A). MT in A549 and MDCK cells are heavily acetylated, with about 70% of α-tubulin residues containing the posttranslational modification, as determined by Western blotting. Acetylated MT are present in HBE cells but are much less abundant, with an acetylated MT to total MT ratio of 0.15 compared to 0.70 and 0.77 in MDCK and A549 cells, respectively (Fig. 4A). Unexpectedly, we observed that nocodazole treatment resulted in a loss of acetylated MT that was independent of virus infection in all of the cell types tested (Fig. 4B and C). Given this observation, we can exclude the possibility that vRNPs travel on acetylated MT in the cytoplasm. Other posttranslational modifications of MT have been reported, including phosphorylation, detyrosination, ubiquitination, and others (36). Detyrosination and glutamylation have been linked to MT stabilization and may alter sensitivity to nocodazole (36). The role of these modifications in influenza virus replication may require further study. Taking these results together with our previous observations on the mislocalization of Rab11A and unperturbed influenza virus replication, we speculate that influenza vRNA segments are packaged into infectious virions independently of acetylated MT filaments.

### vRNA composition of cytoplasmic subcomplexes is maintained in the absence of MT filaments.

**FIG 5:**
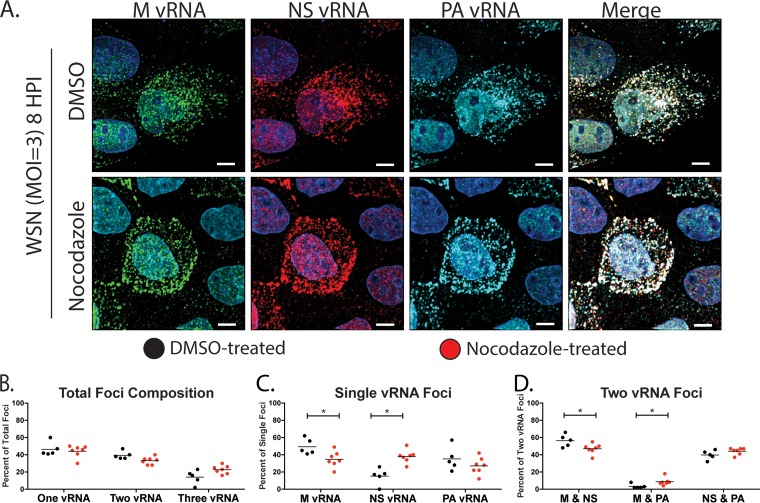
Visualization of multiple vRNA segments in MDCK cells treated with nocodazole. (A) MDCK cells were infected with A/WSN/1933 (H1N1) and treated with DMSO or nocodazole (5 μg/ml) at 4 hpi and fixed at 8 hpi for FISH staining. FISH probes targeting the M vRNA segment (Alexa 488, green), NS vRNA (Quasar 570, red), and PA vRNA (Quasar 670, teal) were used. DAPI (blue) marks the cell nuclei, and the scale bars are 5 μm. Single representative cells chosen from the entire coverslip are displayed for clarity. (B to D) Finely spaced confocal stacks were acquired to reconstruct a 3D cell volume. Deconvolved stacks were analyzed via Imaris software to generate spots corresponding to the individual vRNA segments and to analyze the colocalization of spots. The proportions of foci containing one, two, or all three of the labeled vRNA segments are depicted in panel B. We further present a breakdown of the composition of each focus containing only one (C) or two (D) labeled vRNA segments. Each data point represents an individual cell that was analyzed by this method. A nonparametric Mann-Whitney test was performed to compare foci from DMSO (*n* = 5, black circles)- and nocodazole (*n* = 7, red circles)-treated cells.

**FIG 6:**
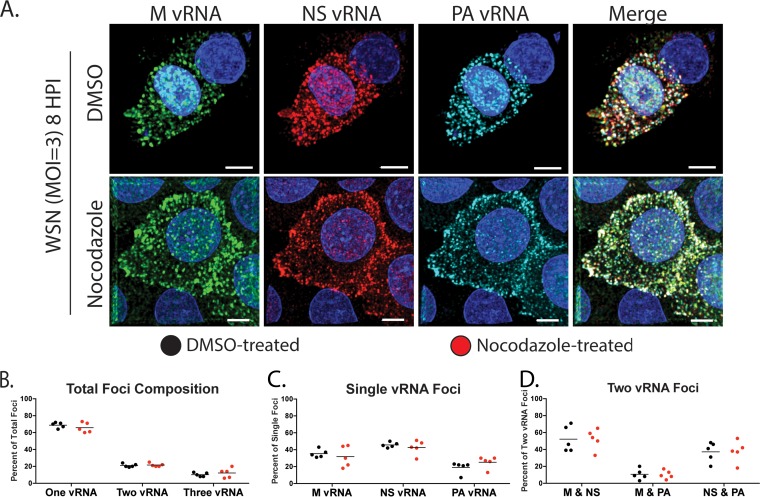
Visualization of multiple vRNA segments in A549 cells treated with nocodazole. (A) A549 cells were infected with A/WSN/1933 (H1N1), treated with DMSO or nocodazole (5 μg/ml) at 4 hpi, and fixed at 8 hpi for FISH staining. FISH probes targeting the M (Alexa 488, green), NS (Quasar 570, red), and PA (Quasar 670, teal) vRNA segments were used. DAPI (blue) marks the cell nucleus, and scale bars are 5 μm. Single representative cells chosen from the entire coverslip are displayed for clarity. (B to D) Fine confocal stacks were acquired to reconstruct a 3D cell volume. Deconvolved stacks were analyzed via Imaris software to generate spots corresponding to the individual vRNA segments and analyze the colocalization of spots. The proportion of foci containing one, two, or all three of the labeled vRNA segments is depicted in panel B. We further present a breakdown of the composition of each focus containing only one (C) or two (D) labeled vRNA segments. Each data point represents an individual cell that was analyzed by this method. A nonparametric Mann-Whitney test was performed to compare foci from DMSO (*n* = 5, black circles)- and nocodazole (*n* = 5, red circles)-treated cells.

We and others have previously demonstrated that vRNA segments are exported from the nucleus as subcomplexes that undergo further assembly en route to the plasma membrane (9, 10). We speculate that dynamic fusion and fission of Rab11A RE facilitate the cytoplasmic assembly of the vRNA subcomplexes. To determine whether MT contribute to the assembly of vRNA subcomplexes, we assessed the composition of cytoplasmic foci by visualizing the localization of three vRNA segments, M, NS, and PA, by FISH. Pairwise analysis of vRNA segments from published multiple four-color FISH experiments (10) revealed that M and PA vRNA segments are not highly associated in the cytoplasm, while NS associates with similar frequencies with both M and PA (S. S. Lakdawala, unpublished data). Therefore, to determine the role of MT in the ordered assembly of vRNA segments, we focused on these three segments by looking at vRNA pairs with low affinity (M and PA) and those with moderate affinity (NS with PA or M). We performed FISH with nocodazole- and DMSO-treated MDCK (Fig. 5) and A549 (Fig. 6) cells. These cells were imaged on a confocal microscope with fine z-stack sampling (0.17-μm step size) to produce 3D images of stained cells. A similar total number of spots was generated in each cell type by treatment with DMSO or nocodazole (Table 1). In general, we found similar vRNA compositions in cytoplasmic foci in DMSO- and nocodazole-treated cells (Fig. 5B to D). We analyzed the proportion of cytoplasmic foci containing one, two, or three of the labeled vRNA segments and observed no significant difference in nocodazole- and DMSO-treated cells (Fig. 5B and 6B). Additionally, analysis of the composition of foci containing a single labeled vRNA revealed that more foci contained only labeled NS vRNA and fewer contained only labeled M vRNA in nocodazole-treated cells than in DMSO-treated cells (Fig. 5C). This result does not imply that the NS segment is transported individually since there are five additional vRNA segments not labeled within the cells. Similarly, investigation of the composition of foci composed of two colocated vRNAs presented a small increase in the association of M and PA in MDCK cells treated with nocodazole (Fig. 5D). These data suggest that some cytoplasmic vRNA-vRNA associations are altered during nocodazole treatment within MDCK cells.

**TABLE 1:** Summary of the total numbers of spots from all of the samples analyzed in this study^*a*^

Expt and condition	Total no. of foci	Total no. of spots	
		Rab11A	vRNP
A549 + WSN at 8 hpi, 3-color FISH			
DMSO	1,186; 1,756; 1,106; 1,447; 1,253		
Nocodazole	1,579; 1,822; 1,457; 1,377; 1,053		
MDCK + WSN at 8 hpi, 3-color FISH			
DMSO	1,311; 1,461; 1,428; 1,243; 1,005		
Nocodazole	1,349; 1,103; 1,516; 819; 1,310; 1,116; 1,616		
A549 Rab11A GFP + WSN 8 at hpi, PA vRNA Rab11A colocalization			
DMSO	548; 555; 509; 459; 1,009; 762	321; 308; 361; 342; 755; 603	385; 357; 312; 334; 563; 442
Nocodazole	1,373; 810; 994; 2,197; 985; 1,044	678; 397; 450; 1,036; 388; 481	866; 592; 634; 1,416; 688; 642
A549 Rab11A GFP + WSN at 8 hpi, NP Rab11A colocalization			
DMSO	596; 867; 701; 543; 607; 373; 350; 320; 444	490; 509; 510; 441; 435; 282; 235; 248; 350	295; 580; 477; 426; 399; 262; 248; 220; 288
Nocodazole	608; 582; 659; 916; 650; 855; 707; 678; 994; 820	424; 431; 404; 487; 484; 540; 400; 501; 634; 528	283; 324; 365; 513; 331; 626; 407; 457; 725; 571
A549 Rab11A GFP + WSN at 8 hpi, NP Rab11A colocalization			
DMSO	771; 1,010; 963; 593; 510	569; 808; 597; 468; 359	562; 798; 674; 344; 311
Paclitaxel	1,569; 409; 710; 613; 1,293; 522; 630	929; 256; 528; 370; 761; 331; 423	740; 157; 372; 254; 556; 213; 371

a Numbers of foci are presented for each cell, and cells are separated by semicolons. The total number of spots includes Rab11A-alone, vRNP-alone. and colocated spots, while the total number of Rab11A spots includes Rab11A-alone and colocated spots. The same is true of the total number of vRNP spots. Therefore, the sum of spots presented in the total number of Rab11A spots and the total number of vRNP spots for a given cell is greater than the total number of foci for that cell, as it includes colocated spots counted twice.

**FIG 7:**
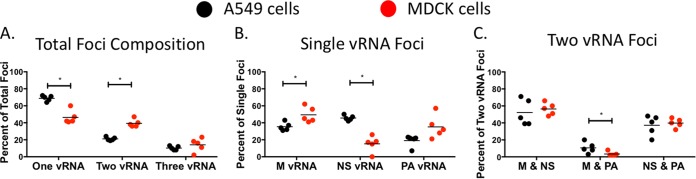
Comparison of vRNA-containing cytoplasmic foci in MDCK and A549 cells. An analysis of the composition of each cytoplasmic focus from MDCK and A549 cells treated with DMSO conducted as shown in Fig. 5 and 6 is presented for direct comparison. The proportion of foci containing one, two, or all three of the labeled vRNA segments is depicted in panel A. We further present a breakdown of the composition for each focus containing only one (B) or two (C) labeled vRNA segments. Each data point represents an individual cell that was analyzed by this method. A nonparametric Mann-Whitney test was performed to compare foci from A549 (*n* = 5, black circles) and MDCK (*n* = 5, red circles) cells.

To assess the robustness of this result, we carried out similar experiments with A549 cells (Fig. 6). As expected, the overall compositions of vRNA-containing foci were similar in nocodazole- and DMSO-treated cells (Fig. 6B). Surprisingly, we observed similar compositions of foci containing one or two labeled vRNA segments in DMSO- and nocodazole-treated A549 cells (Fig. 6B to D). These observations suggest that in A549 cells, MT do not exclusively mediate the assembly of multiple vRNA segments in the cytoplasm. In addition, the data from A549 and MDCK cells imply that the vRNA composition of cytoplasmic foci may differ with the cell type. To highlight this, we compared cytoplasmic vRNA subcomplexes in A549 and MDCK cells treated with DMSO (Fig. 7). The proportion of foci containing only one of the three labeled vRNAs was significantly higher in A549 cells, while that of foci containing two of three vRNAs was significantly higher in MDCK cells. Further breakdown of the foci containing only single labeled vRNA segments showed an increase in foci containing the M vRNA in MDCK cells, while the percentage of foci containing NS vRNA was increased in A549 cells. On the basis of our previously published data on MDCK cells (10), M and PA vRNA segments have a low cytoplasmic association, which is consistent with results from this study that found a significantly lower M and PA association in MDCK cells than in A549 cells. Taken together, these observations suggest that the cytoplasmic association between vRNA segments may be dependent on the host cell type and further study in physiologically relevant cell culture models is needed to determine the precise order of vRNA assembly. In addition, the cellular morphology may impact our assessment of vRNP colocalization within the cytoplasm.

### Association of Rab11A and vRNP segments is MT dependent.

**FIG 8:**
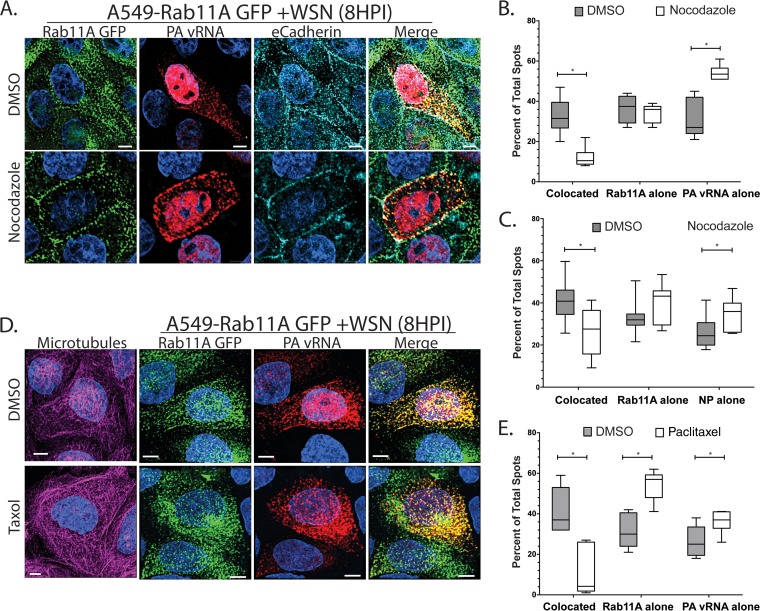
MT promote the cytoplasmic colocalization of Rab11A and IAV vRNA. (A) A549 cells stably expressing GFP-tagged Rab11A (A549-GFP) were infected with A/WSN/1933 (H1N1) at an MOI of 3 and treated with DMSO or nocodazole (5 μg/ml) at 4 hpi. Cells were stained at 8 hpi by FISH against the PA vRNA segment (Quasar 670, red). Additionally, cells were stained by immunofluorescence for E-cadherin (teal) to outline the location of the plasma membrane. Images represent a single confocal slice from samples where finely spaced z stacks were acquired to determine the 3D localization of each protein. DAPI (blue) marks the cellular nucleus, and scale bars are 5 μm. Single representative cells chosen from the entire coverslip are displayed for clarity. (B) Deconvolved stacks were analyzed with Imaris software to generate spots corresponding to Rab11A and vRNA segments. Quantification of the cytoplasmic foci that contain both Rab11A and PA vRNA (colocated) and noncolocated spots (Rab11A alone, PA vRNA alone) was done with Imaris. The proportions of the total spots in each cell that were colocated, Rab11A-alone, or vRNP-alone spots are presented for DMSO-treated (*n* = 6) and nocodazole-treated cells (*n* = 6) as a box-and-whisker plot. A nonparametric Mann-Whitney test was performed to compare foci from DMSO- and nocodazole-treated cells. (C) A549 Rab11A-GFP cells were infected and treated with nocodazole as described for panel A and then stained with antibodies against E-cadherin and viral NP. The proportions of colocated and noncolocated foci are displayed for DMSO (*n* = 9)- and nocodazole (*n* = 10)-treated cells as a box-and-whisker plot. (D, E) A549 Rab11A-GFP cells were treated with paclitaxel at 4 hpi with WT WSN virus. Colocalization of Rab11A and PA vRNA was determined as described above, and a comparison of colocated and noncolocated spots in DMSO (*n* = 5)- and paclitaxel (*n* = 5)-treated cells is presented.

We have demonstrated that Rab11A and vRNP segments are redistributed in the absence of MT filaments; however, the degree of relocalization between Rab11A and vRNP is not equivalent (Fig. 1). Therefore, we examined whether the proportion of vRNP segments associated with Rab11A GFP is dependent upon the presence of intact MT filaments. We calculated the proportion of cytoplasmic foci containing both vRNA (specifically, PA vRNA) and Rab11A in A549 Rab11A-GFP cells infected with WSN and treated with DMSO or nocodazole (Fig. 8A). A significant reduction in colocated spots (those containing both Rab11A-GFP and the PA vRNA segment) in nocodazole-treated cells was observed (Fig. 8B). This reduction in colocated spots correlated with a significant increase in foci containing only PA vRNA. To confirm that this phenomenon was not unique to PA vRNA, we performed similar experiments using immunofluorescence for NP rather than FISH staining of a single vRNA segment. Convincingly, we observed that colocalization of NP and Rab11A was also dependent upon the presence of intact MT filaments (Fig. 8C). These observations suggest that association of Rab11A and vRNP segments is dependent on intact MT. This particular analysis method is dependent upon the total number of foci per cell. Table 1 lists the total number of foci, as well as the total number of Rab11A and vRNP spots, per cell to compare whether nocodazole treatment impacts the number of vRNP and Rab11A spots observed within a cell. The total numbers of foci and Rab11A and vRNP spots were consistent in DMSO- and nocodazole-treated cells, demonstrating that the decrease in colocated spots was not based on differences in the number of foci within a cell. We also tested whether treatment with an MT-stabilizing drug (paclitaxel [originally named taxol]) would abrogate the association of Rab11A and NP. A549 cells stably expressing Rab11A-GFP were treated with paclitaxel (5 μM) from 4 to 8 hpi, at which point the cells were fixed and stained (Fig. 8D). We analyzed the colocalization of PA vRNA and Rab11A-GFP within the paclitaxel-treated cells and compared it to that in DMSO-treated cells. We found that treatment of cells with paclitaxel reduced the colocalization of Rab11A and PA vRNA similar to that in cells treated with nocodazole (Fig. 8E). These data suggest that dynamic MT filaments are necessary for the association of Rab11A and vRNP segments.

**FIG 9:**
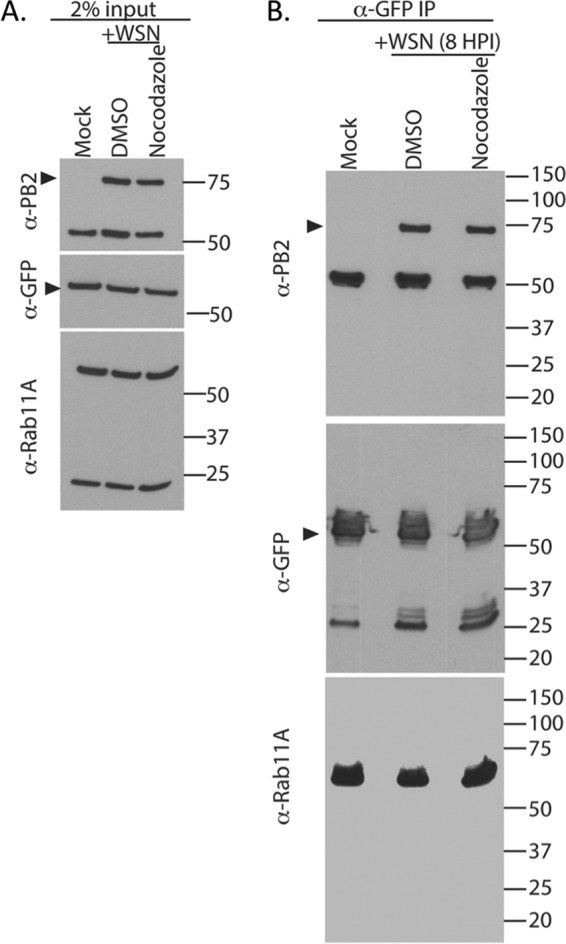
Pulldown of Rab11A-GFP in nocodazole-treated cells shows no difference in bound PB2 levels. A549 Rab11A-GFP cells were infected with WT WSN (MOI of 3) and treated with DMSO or nocodazole, and the association between Rab11A-GFP and PB2 was determined at 8 hpi. (A) The 2% input displays equal levels of Rab11A-GFP in infected and uninfected cells, as well as equal levels of PB2 in DMSO- and nocodazole-treated cells. (B) IP with anti-GFP antibody-conjugated beads demonstrates equal levels of immunoprecipitated (IP) GFP and Rab11A, as well as similar levels of bound PB2 within an entire population of cells. Dark arrowheads mark the correct band for PB2 and Rab11A-GFP in the anti-PB2- and anti-GFP-specific blots. The values to the right are molecular sizes in kilodaltons.

To expand upon this observation, we performed an immunoprecipitation (IP) experiment with infected A549 Rab11A-GFP cells to assess whether we could observe a decrease in the global association of PB2 and Rab11A upon nocodazole treatment (Fig. 9). A549 Rab11A-GFP cells were infected with WSN, and at 4 hpi, the cells were treated with DMSO or nocodazole, similar to the studies presented in Fig. 8. At 8 hpi, the cells were washed and harvested for IP with anti-GFP-specific beads. As evident in the input, the levels of PB2 were similar in DMSO- and nocodazole-treated cells (Fig. 9A). Additionally, the levels of exogenous Rab11A-GFP (∼55 kDa) were similar to the levels of endogenous Rab11A (∼24 kDa) (Fig. 9A). IP with GFP pulled down equivalent amounts of PB2 in infected samples treated with DMSO or nocodazole (Fig. 9B). The levels of GFP-tagged Rab11A pulled down in all of the samples were equal, confirming the efficiency of pulldown (Fig. 9B). These data suggest that there is a substantial proportion of Rab11A and PB2 associated within a cell during nocodazole treatment, such that when all cells are aggregated in an IP assay, subtle differences are obscured. However, further analysis of the specific localization of Rab11A and vRNP colocalization within individual cells may help to tease apart observations made at the cellular level.

### Defining the intracellular location of Rab11A and influenza vRNP association.

**FIG 10:**
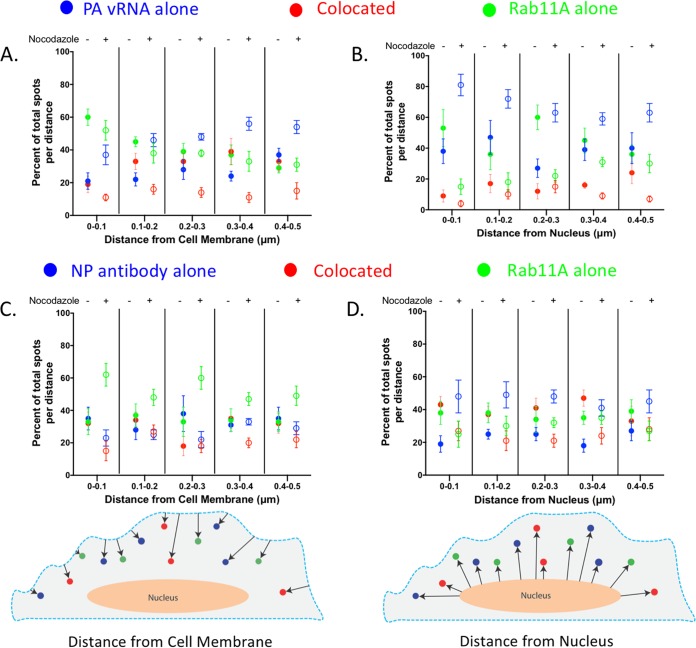
Cellular distribution of Rab11A and vRNP colocated and noncolocated spots is disrupted in the absence of MT. (A to D) Data from A549 Rab11A-GFP cells presented in Fig. 8 were further analyzed for the distribution of foci at various distances from the nucleus and plasma membrane. DAPI staining was used to define the nuclear volume, while E-cadherin was used to mark the cellular membrane of all of the 3D cell volumes. Using Imaris, we were able to determine the distances between the spots generated and the cell membrane (A and C) and the nucleus (B and D). Shown here is the proportion of each type of foci, i.e., colocated (red), PA vRNA or NP alone (blue), and Rab11A alone (green), in the total number of spots at that specific distance. DMSO-treated cells are shown as solid circles, and nocodazole-treated cells are shown as open circles. Cell schematics are included to highlight how the distance measurements were generated, the dashed blue line represents the E-cadherin signal, and the nucleus (peach) was generated from the DAPI signal.

To define where Rab11A-GFP and PA vRNA are colocated inside the cell, we determined the number of cytoplasmic foci found within 100-nm-wide bins for the first 500 nm from both the plasma membrane and the nucleus that contained Rab11A alone, colocated Rab11A and vRNP, or vRNP alone (Fig. 10). The vRNPs were visualized by either PA vRNA FISH (Fig. 10A and B) or anti-NP immunostaining (Fig. 10C and D). The nuclear boundary was defined by a 4′,6-diamidino-2-phenylindole (DAPI) signal, and E-cadherin staining was used to define the plasma membrane. In DMSO-treated cells, we found that within 100 nm of the plasma membrane, only 20% of the foci contain both Rab11A and PA vRNA (shown as colocated foci), but that this proportion increased to 40% between 300 and 400 nm from the plasma membrane before reducing slightly further away (Fig. 10A, red solid circles). Similarly, we observed that at 500 nm from the plasma membrane, 40% of the Rab11A-GFP and NP spots were colocated (Fig. 10C). Interestingly, in nocodazole-treated cells, the proportion of colocated spots remained around 20% of the total foci at all distances from the plasma membrane in cells costained with NP and less than 10% in cells costained with PA vRNP (Fig. 10A and C, red open circles). In contrast, the proportion of foci containing only PA-vRNA increased with distance from the plasma membrane in cells treated with nocodazole (Fig. 10A, open blue circles). On the basis of the redistribution of Rab11A-GFP to the plasma membrane in nocodazole-treated cells (Fig. 1A), we had expected that Rab11A-containing foci would be observed only at the plasma membrane. Interestingly, in nocodazole-treated cells, between 40 and 60% of the total foci at each distance contained Rab11A-GFP alone, although the proportion of Rab11A was not as consistent between PA and NP vRNA-stained cells, the trend is consistent with the redistribution observed by immunofluorescence (Fig. 10A and C, open green circles). Taken together, our data suggest that Rab11A can be found inside the cell in the absence of MT but is no longer associated with vRNA. It is unclear whether the Rab11A-only cytoplasmic foci represent free Rab11A proteins or if they remain associated with endosomes.

Analysis of the composition of foci at various radial distances from the nucleus was also performed (Fig. 10B and D). In DMSO-treated cells, we observed that Rab11A and PA vRNA were colocated in about 10% of the foci within 400 nm of the DAPI signal, but this increased to 30% between 400 and 500 nm from the nucleus (solid red circles). This percentage continued to increase at greater distances from the nucleus, suggesting that 400 to 500 nm might be where the vRNA segments associate with Rab11A RE. In contrast, colocalization of NP and Rab11A was found to be around 40% at the nuclear periphery and was a similar percentage until 500 nm from the nuclear boundary (Fig. 10D). The discrepancy between Rab11A colocalization with PA vRNA compared to NP is expected because some Rab11A vesicles would be associated with other vRNP segments, just not with PA vRNA.

In the presence of nocodazole treatment, the proportion of colocated Rab11A and vRNP (either PA or NP) at any distance from the nucleus was lower than that in DMSO-treated cells (Fig. 10B and D, open and solid red circles). In addition, we observed that most of the foci at all radial distances from the nucleus in nocodazole-treated cells contained either PA vRNA or NP alone (open blue circles, Fig. 10B and D). These data demonstrate that colocated Rab11A and vRNP foci are reduced throughout the cytoplasm in nocodazole-treated cells, which is consistent with our analysis of the overall composition of the cytoplasmic foci (Fig. 8B and C).

**FIG 11:**
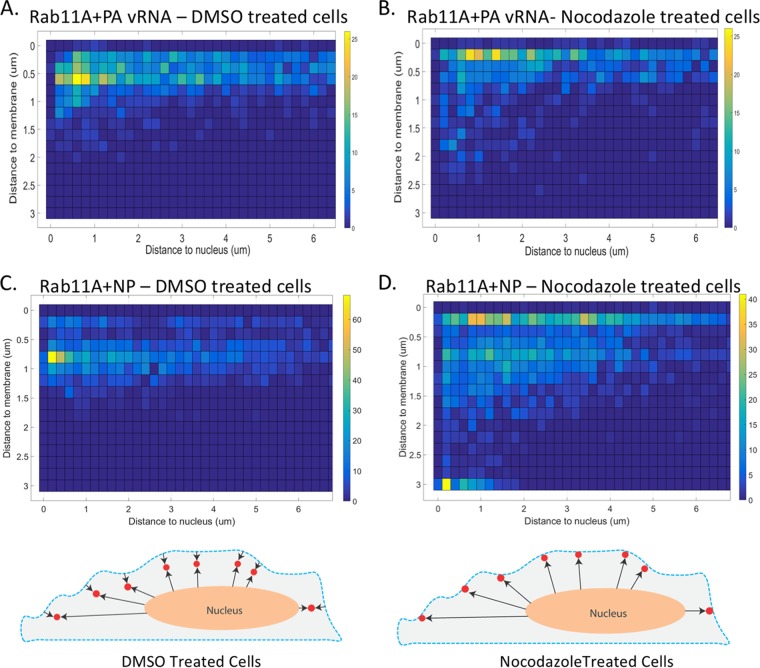
Intracellular distribution of Rab11A GFP and PA vRNA colocated spots is dependent upon MT. Detailed analysis of colocated foci in A549 Rab11A-GFP cells was done to determine where, within the cell, the association and disassociation of Rab11A and vRNA occur. (A, B) 2D heat maps depicting the frequency of Rab11A and NP colocated spots classified by their distances from the nucleus and the plasma membrane within DMSO (*n* = 9)- and nocodazole (*n* = 10)-treated cells. (C, D) Heat maps depicting the frequency of Rab11A-GFP and PA vRNA colocated spots classified by their distances from the nucleus and the plasma membrane within DMSO (*n* = 6)- and nocodazole (*n* = 6)-treated cells. Yellow indicates a large proportion of the foci, while blue is a lack of colocated foci at that position. Each box represents 200 nm on the *x* and *y* axes. Cell schematics are included to highlight the difference in Rab11A and vRNP colocated spots (red) in DMSO- and nocodazole-treated cells. The dashed blue line represents the E-cadherin signal, and the nucleus (peach) was generated from the DAPI signal.

Cells are not spherical; thus, it is difficult to assess where, within a cell, Rab11A and vRNA associate by looking at only the distances to the plasma membrane and nucleus individually. Therefore, to determine the intracellular locations of Rab11A and vRNP where association and disassociation occur, we analyzed colocated foci as a function of their distance from the plasma membrane, as well as the nucleus, in DMSO- and nocodazole-treated cells (Fig. 11). In DMSO-treated cells (*n* = 6), we observed that most colocated foci (green- to yellow-shaded boxes) were located at ∼500 nm from the cell membrane and within 200 to 600 nm of the cell nucleus (Fig. 11A and C). In cells treated with nocodazole (*n* = 6), we observed that most colocated foci (green- to yellow-shaded boxes) were located within 300 nm of the cell membrane and between 700 and 1,600 nm from the nucleus (Fig. 11B and D). Data from DMSO-treated cells suggest that Rab11A and viral NP associate within 200 to 500 nm of the nuclear surface and disassociate at around 600 nm from the plasma membrane. However, in nocodazole-treated cells, Rab11A and vRNP remain associated at or within 200 nm of the plasma membrane. These observations support a model where vRNP segments associate with Rab11A at ∼300 nm from the nucleus and disassociate ∼500 nm from the plasma membrane.

## DISCUSSION

Assembly of influenza vRNA is a highly coordinated process where many of the players and mechanisms are unknown. Our work explores the importance of MT in vRNA assembly and expands upon previously published data implicating MT and Rab11A in vRNP cytoplasmic transport. In this study, we demonstrate that Rab11A and vRNP colocalization requires the presence of intact MT filaments (Fig. 8), yet the absence of MT filaments does not adversely impact viral replication (Fig. 2) (25). We further provide a comprehensive analysis of vRNA and Rab11A intracellular colocalization, detailing where their association occurs within a cell (Fig. 10 and 11). In addition, we observed that the composition of cytoplasmic vRNA subcomplexes varies between cell types (Fig. 5 to 7), suggesting that host factors may impact the vRNA segment assembly process. Taken together, our observations highlight the complex nature of vRNP assembly and support the presence of cell type-dependent and Rab11A-independent mechanisms of vRNA transport and assembly. These data contribute to our understanding of the vRNA transport process from the nucleus to the plasma membrane.

We have previously proposed a model where the dynamic fusion and fission of endosomes promote the assembly of all eight vRNP segments (10). In this study, we used multicolor FISH to explore the cytoplasmic relationship of three vRNA segments in cells with or without intact MT filaments (Fig. 5 and 6) to assess whether MT promote the assembly of all eight vRNP segments. Our investigation of the composition of cytoplasmic foci containing labeled vRNA revealed that nocodazole treatment altered the composition of cytoplasmic foci in MDCK cells but not in A549 cells. The alteration of vRNA association in the cytoplasm of MDCK implies that MT may be required for the dynamic movement of Rab11A endosomes in this cell type. Importantly, we observed differences in the vRNA composition of cytoplasmic foci between MDCK and A549 cells, suggesting that the composition and order of vRNA assembly may be cell type specific. These differences may be related to delays in replication kinetics observed in these two cell types (Fig. 2); therefore, additional studies exploring the intracellular vRNA association at different time points and using multiple cell culture models, including differentiated HBE cells, are needed.

Our ability to generate 3D renderings of infected cells allows for refined spatial analysis of the intracellular colocalization of Rab11A and vRNP. These data increase our understanding of how vRNPs disassociate from Rab11A prior to packaging. Given that Rab11A has not been observed in purified virions (37, 38), there are two potential mechanisms to explain the release of vRNP from Rab11A RE; i.e., (i) Rab11A RE-containing vRNP segments fuse to the plasma membrane and the vRNPs are transferred to budding sites at the plasma membrane, or (ii) vRNP segments are pulled off Rab11A RE at a certain distance from the plasma membrane at influenza virus budding sites. We observed that a large proportion of Rab11A and vRNP spots were colocated 500 nm from the plasma membrane (Fig. 10). This result supports a model where vRNA segments disassociate from Rab11A RE away from the plasma membrane and move to viral budding sites.

Our analysis of cells treated with nocodazole revealed that MT are important for cytoplasmic association of Rab11A and vRNP (Fig. 8 and 10) but are not essential for virus replication in a variety of cell culture models (Fig. 2 and 3). These data suggest a potential Rab11A-independent vRNP transport process. However, studies using small interfering RNA or dominant negative Rab11A constructs have observed a robust and significant reduction in viral titers (13, 29), suggesting that the limited association of Rab11A and vRNP in the cytoplasm of nocodazole-treated cells may be sufficient to efficiently produce progeny virions. Additionally, it is possible that the cell morphology is altered upon nocodazole treatment, which could impact where the colocalization of Rab11A and vRNP is observed in our studies. However, we did not observe any significant variations in cell thickness during our experiments. Interestingly, recent live imaging analysis of the cellular organelle interactome using a lattice light-sheet microscope revealed that nocodazole treatment did not greatly affect the interplay of intracellular lipid droplets, such as endosomes, with other cellular features such as the endoplasmic reticulum and Golgi apparatus (39). Therefore, nocodazole treatment does not shut down all intracellular dynamics and further work studying the relationship of vRNP and cellular organelles could help to elucidate alternative vRNP transport pathways.

The results presented here enhance our understanding of how MT impact influenza vRNA cytoplasmic transport and assembly. Further investigation of the transport of Rab11A and vRNP in live cells during a productive infection is needed and will help provide further information on the dynamics of vRNA assembly and the presence of a possible Rab11A-independent transport mechanism.

## MATERIALS AND METHODS

### Cells and viruses.

MDCK epithelial cells obtained from ATCC and type II MDCK cells provided by Ora Weisz (University of Pittsburgh School of Medicine) were maintained in modified Eagle's medium (MEM) with 10% fetal bovine serum (FBS) and l-glutamine. A549 cells (ATCC) were maintained in Dulbecco's MEM supplemented with 10% FBS. Fully differentiated HBE cells were obtained from the University of Pittsburgh airway epithelial cell core facility through an Institutional Review Board-approved protocol. HBE cells were grown on transwells at an air-liquid interface and prepared and cultured as previously described (40).

Recombinant A/WSN/1933 (WSN) virus was rescued as previously described (10). Recombinant pandemic H1N1 (A/California/07/2009) virus was generated as previously described (41, 42), while the A/Perth/16/2009 H3N2 virus was a generous gift from Zhiping Ye (Center for Biologics Evaluation and Research, FDA).

A549 cells stably expressing Rab11A-GFP were generated by transfection of a Rab11A-GFP expression plasmid, a generous gift from Ora Weisz (University of Pittsburgh), by using TransIT-LT1 transfection reagent (Mirus Bio LLC) in accordance with the manufacturer's protocol. Clonal populations of cells were isolated by plating an average of a single cell per well into 96-well plates and expanding clones that tested positive for Rab11A-GFP expression under selection with 1,000 μg/ml G418 sulfate.

### IAV infection and replication kinetics.

A549 and MDCK cells were inoculated with the virus indicated at an MOI of 0.01 (calculated on the basis of the 50% tissue culture infective dose [TCID_50_] or as otherwise specified in the figure legends) in MEM containing 2% l-glutamine and supplemented with l-(tosylamido-2-phenyl) ethyl chloromethyl ketone (TPCK)-treated trypsin (Worthington Biochemical). After 1 h of incubation, the virus inoculum was removed and the cells were washed, overlaid with fresh medium, and incubated at 37°C. At 4 hpi, the medium was replaced with fresh medium containing DMSO (1:1,000) or 5 μg/ml (or 16 μM) nocodazole for the duration of the infection. This concentration of nocodazole is within the range of concentrations used to depolymerize MT in MDCK cells to study influenza virus or intracellular transport of cargo; the concentrations used have ranged from 2 to 33 μM (11, 20, 25, 34, 43–49). In A549 cells, studies have used nocodazole concentrations of 0.1 to 20 μM (50–54). Supernatants from infected cells were harvested at various times ranging from 6 to 72 hpi for viral titration. Virus titers of the collected supernatants were measured by TCID_50_ determination by the endpoint method of Reed and Muench (55) and expressed as log_10_ TCID_50_s per milliliter. The experiments were done in triplicate with at least two biological replicates. The viability of cells treated with nocodazole was determined with a fluorescence assay where treated cells were stained with Hoechst, a live nuclear stain, and propidium iodide (PI) at 6, 8, and 24 h posttreatment and compared with that of DMSO-treated cells. More than 5,000 cells were assayed per condition per time point by taking a large-field-of-view (×10 magnification) image. Imaris was used to quantify the numbers of Hoechst-positive and PI-negative cells, which were live cells, and Hoechst and PI double-positive cells, which were dead cells. The percentage of cell survival was calculated as the ratio of live cells (Hoechst positive, PI negative) to the total number of cells counted (all Hoechst-positive cells). Minimal loss of cell viability was observed at 24 h posttreatment, and no loss of cell survival was observed at earlier time points.

Replication of influenza viruses in HBE cells in transwells was analyzed as follows. The apical surface of cells was washed in phosphate-buffered saline (PBS), and then 10^3^ TCID_50_s of virus was added per 100 μl of HBE growth medium. After incubation for 1 h at room temperature with gentle rocking, the apical surface of cells was washed three times with PBS. The final wash was collected as the 1-h time point. At 24 hpi, 200 μl of HBE medium supplemented with 0.05% bovine serum albumin (BSA) was added to the apical surface for 15 min to capture released virus particles. After virus collection at 24 hpi, HBE medium containing DMSO (1:500) or 10 μg/ml nocodazole was placed on the apical (100 μl) and basal surfaces (500 μl). The medium was maintained for 24 h. The transepithelial electrical resistance of the cells was determined with an Epithelial Volt Ohm Meter (World Precision Instruments) before and after treatment, and no change was observed over the 24 h, indicating that polarity was maintained at ∼1,000 Ω. At 24 h posttreatment (and 48 hpi), the medium was collected from the apical side for virus titration. The experiment was done in triplicate, and at least three different patient cell lines were tested.

### Western blotting.

MDCK and A549 cells grown on six-well plates, and HBE cells on transwells were lysed in lysis buffer (1% Triton X-100, 10% glycerol, 25 mM HEPES) on ice. A 50-μg sample of protein lysate, as determined by Bradford assay (Thermo-Fisher Scientific), was separated by SDS–4 to 12% morpholineethanesulfonic acid PAGE (Invitrogen). The proteins were transferred to polyvinylidene difluoride membranes (Bio-Rad) and blocked in 5% milk in PBS with 0.1% Tween 20. The membranes were incubated with the primary antibodies (mouse anti-acetylated α-tubulin monoclonal antibody [purchased from Sigma] at 1:1,000, rabbit anti-α-tubulin monoclonal antibody [from Cell Signaling] at 1:1,000) in 3% BSA and 0.04% sodium azide in PBS overnight at 4°C. The membranes were subsequently probed with horseradish peroxidase (HRP)-conjugated anti-mouse or anti-rabbit IgG antibodies (Bio-Rad) in 5% milk and visualized with enhanced chemiluminescence reagents (Bio-Rad).

### IP.

Ten-centimeter dishes of A549 Rab11A-GFP cells were infected with wild-type (WT) WSN (MOI of 3) and then treated with DMSO or nocodazole (5 μg/ml) at 4 hpi. Cells were washed, harvested at 8 hpi, and centrifuged at a low speed. Lysates were generated by resuspending the cell pellet from a single 10-cm dish into 1 ml of PXL buffer (1× PBS, 1% NP-40, 0.5% deoxycholate, 0.1% SDS). Anti-GFP Dynabeads from MBL International Corporation were used in accordance with the manufacturer's recommendation. IP of 250 μl of lysate per condition was performed with 50 μl of anti-GFP beads at 4°C for 5 h. Beads were washed three times with PXL buffer and subjected to one high-salt wash (200 nM NaCl, 300 nM sodium acetate). Samples were then boiled and subjected to SDS-PAGE. Western blotting with anti-PB2 (BEI number NR-50356), anti-Rab11A (Abcam ab65200), and anti-GFP (rabbit; Thermo Fisher Scientific) antibodies was performed. HRP-conjugated secondary antibodies (Jackson Laboratories) with minimal cross-reactivity to rat serum were used for detection.

### Immunofluorescence.

A549 and MDCK cells seeded onto glass coverslips were infected with WSN at an MOI of 3 for the times indicated in the respective figure legends. Cells were fixed in 4% paraformaldehyde for visualization of most viral and host proteins, except MT, which were visualized in cells fixed with 100% methanol at −20°C for 3 min. Briefly, cells were permeabilized in 0.5% Triton X-100 and blocked with 3% BSA. The cells were subsequently incubated with the antibodies. Finally, the cells were treated with the DNA stain DAPI to visualize the nuclear volume.

Primary antibodies were purchased commercially and used at the following dilutions: anti-acetylated alpha-tubulin antibody (Sigma), 1:1,000; anti-alpha-tubulin antibody (Sigma), 1:2,000; anti-E-cadherin antibody (Cell Signaling), 1:100. The anti-NP primary antibody used (HB-65 at 1:1,000) was a kind gift from Jon Yewdell (NIAID). Alexa Fluor 488-, Alexa Fluor 594-, and Alexa Fluor 647-conjugated anti-mouse and anti-rabbit F(ab′)_2_ fragments (Invitrogen) were used for visualization.

### FISH.

Infected cells were analyzed by FISH as described previously (10). For these studies, we utilized FISH probes against WSN gene segments M, NS, and PA conjugated to Alexa Fluor 488, Quasar 570, and Quasar 670, respectively. The oligonucleotides used were obtained from Biosearch Technologies. The fluorophores were visualized with an Olympus FluoView FV1000 confocal microscope with a 60× oil immersion objective. Stacks of each cell were taken at z intervals of 0.17 μm, and FISH staining was used to determine the vertical extent of the cell. These imaging parameters ensured a voxel spacing of approximately 50 by 50 by 170 nm. Spectral separation was confirmed with single-color controls, i.e., staining of infected and fixed cells with a single FISH probe. Probe specificity was determined previously (10).

### Image analysis.

All imaging experiments were repeated at least twice, and a minimum of five cells were analyzed per replicate. Representative cells were selected from a random field of view on the coverslip. Whenever possible, different personnel were responsible for image capturing versus image analysis to avoid any inherent bias. In addition, we have automated as much as possible of our image analysis pipeline to ensure uniformity among all imaging processes.

3D confocal stacks of FISH and immunofluorescence were background subtracted and deconvolved with Huygens Professional (version 16.05; Scientific Volume Imaging B.V.). Deconvolution was done automatically at 40 iterations per deconvolution assuming a signal-to-noise ratio of 20. All images were deconvolved in a similar manner to ensure that no bias in image analysis was introduced.

The resulting images were analyzed with Imaris software (version 8.4.1; Bitplane AG). Imaris was used to generate spots for the GFP-tagged Rab11A foci, viral NP, and FISH probes with the program's “Spots” feature. A threshold of 2× the mean fluorescence intensity standard deviation provided by Imaris for each channel was used for all spot generations to provide an unbiased approach.

To create and quantify the spots, we first defined the cellular and nuclear spaces for a cell of interest. We used the DAPI channel to automatically define the cell nuclear voxel space with a smoothing value of 0.09. A combination of the FISH probes or E-cadherin was used to define the cellular membrane, which was drawn manually on each slice. To determine the proportion of cytoplasmic foci within a cell and their respective distances from the nucleus and plasma membrane, we used a feature of Imaris known as “Cell.” In this feature, the cell contour is defined as the surface on the basis of E-cadherin staining and the nucleus is based on the surface from DAPI staining. To determine the composition of the cytoplasmic foci, we modified the Imaris MatLab extension program called “Colocalization of Spots” to accommodate the comparison of up to four different spots. The revised code is called “XTSpotsColocalizeFISH4.m” and can be found at https://github.com/Lakdawala-Lab/MatLab-Extensions. Using this MatLab extension, we identified colocalized spots by using a distance threshold of 300 nm (the size of our diffraction limit pixel size) from each other. The two-vRNA- and three-vRNA-colocated and noncolocated (single-vRNA-containing) spots were imported into the Cell feature as vesicles, and the number and intracellular location of each spot were exported by using the “statistics” feature of Imaris. The frequency and location data generated by Imaris were graphed and compared by using Prism (version 6.0; GraphPad). Mann-Whitney analysis was used to determine the significance of differences between the data sets from DMSO- and nocodazole-treated samples. Heat maps indicating spot frequency from the nucleus and plasma membrane were plotted by using MatLab from the Imaris-generated data.

## References

[1] Fields BN, Knipe DM, Howley PM 2013 Fields virology, 6th ed Wolters Kluwer/Lippincott Williams & Wilkins Health, Philadelphia, PA.

[2] Noda T, Sagara H, Yen A, Takada A, Kida H, Cheng RH, Kawaoka Y 2006 Architecture of ribonucleoprotein complexes in influenza A virus particles. Nature 439:490–492. doi:10.1038/nature04378. 16437116

[3] Eisfeld AJ, Neumann G, Kawaoka Y 2015 At the centre: influenza A virus ribonucleoproteins. Nat Rev Microbiol 13:28–41. doi:10.1038/nrmicro3367. 25417656 PMC5619696

[4] Fujii Y, Goto H, Watanabe T, Yoshida T, Kawaoka Y 2003 Selective incorporation of influenza virus RNA segments into virions. Proc Natl Acad Sci U S A 100:2002–2007. doi:10.1073/pnas.0437772100. 12574509 PMC149948

[5] Odagiri T, Tashiro M 1997 Segment-specific noncoding sequences of the influenza virus genome RNA are involved in the specific competition between defective interfering RNA and its progenitor RNA segment at the virion assembly step. J Virol 71:2138–2145. 9032347 10.1128/jvi.71.3.2138-2145.1997 PMC191316

[6] Noda T, Sugita Y, Aoyama K, Hirase A, Kawakami E, Miyazawa A, Sagara H, Kawaoka Y 2012 Three-dimensional analysis of ribonucleoprotein complexes in influenza A virus. Nat Commun 3:639. doi:10.1038/ncomms1647. 22273677 PMC3272569

[7] Fournier E, Moules V, Essere B, Paillart JC, Sirbat JD, Isel C, Cavalier A, Rolland JP, Thomas D, Lina B, Marquet R 2012 A supramolecular assembly formed by influenza A virus genomic RNA segments. Nucleic Acids Res 40:2197–2209. doi:10.1093/nar/gkr985. 22075989 PMC3300030

[8] Fournier E, Moules V, Essere B, Paillart JC, Sirbat JD, Cavalier A, Rolland JP, Thomas D, Lina B, Isel C, Marquet R 2012 Interaction network linking the human H3N2 influenza A virus genomic RNA segments. Vaccine 30:7359–7367. doi:10.1016/j.vaccine.2012.09.079. 23063835

[9] Chou YY, Heaton NS, Gao Q, Palese P, Singer R, Lionnet T 2013 Colocalization of different influenza viral RNA segments in the cytoplasm before viral budding as shown by single-molecule sensitivity FISH analysis. PLoS Pathog 9:e1003358. doi:10.1371/journal.ppat.1003358. 23671419 PMC3649991

[10] Lakdawala SS, Wu Y, Wawrzusin P, Kabat J, Broadbent AJ, Lamirande EW, Fodor E, Altan-Bonnet N, Shroff H, Subbarao K 2014 Influenza A virus assembly intermediates fuse in the cytoplasm. PLoS Pathog 10:e1003971. doi:10.1371/journal.ppat.1003971. 24603687 PMC3946384

[11] Amorim MJ, Bruce EA, Read EK, Foeglein A, Mahen R, Stuart AD, Digard P 2011 A Rab11- and microtubule-dependent mechanism for cytoplasmic transport of influenza A virus viral RNA. J Virol 85:4143–4156. doi:10.1128/JVI.02606-10. 21307188 PMC3126276

[12] Bruce EA, Stuart A, McCaffrey MW, Digard P 2012 Role of the Rab11 pathway in negative-strand virus assembly. Biochem Soc Trans 40:1409–1415. doi:10.1042/BST20120166. 23176490

[13] Eisfeld AJ, Kawakami E, Watanabe T, Neumann G, Kawaoka Y 2011 RAB11A is essential for transport of the influenza virus genome to the plasma membrane. J Virol 85:6117–6126. doi:10.1128/JVI.00378-11. 21525351 PMC3126513

[14] Momose F, Sekimoto T, Ohkura T, Jo S, Kawaguchi A, Nagata K, Morikawa Y 2011 Apical transport of influenza A virus ribonucleoprotein requires Rab11-positive recycling endosome. PLoS One 6:e21123. doi:10.1371/journal.pone.0021123. 21731653 PMC3120830

[15] Welz T, Wellbourne-Wood J, Kerkhoff E 2014 Orchestration of cell surface proteins by Rab11. Trends Cell Biol 24:407–415. doi:10.1016/j.tcb.2014.02.004. 24675420

[16] Weisz OA, Rodriguez-Boulan E 2009 Apical trafficking in epithelial cells: signals, clusters and motors. J Cell Sci 122:4253–4266. doi:10.1242/jcs.032615. 19923269 PMC2779128

[17] Takahashi S, Kubo K, Waguri S, Yabashi A, Shin HW, Katoh Y, Nakayama K 2012 Rab11 regulates exocytosis of recycling vesicles at the plasma membrane. J Cell Sci 125:4049–4057. doi:10.1242/jcs.102913. 22685325

[18] Schonteich E, Wilson GM, Burden J, Hopkins CR, Anderson K, Goldenring JR, Prekeris R 2008 The Rip11/Rab11-FIP5 and kinesin II complex regulates endocytic protein recycling. J Cell Sci 121:3824–3833. doi:10.1242/jcs.032441. 18957512 PMC4365997

[19] Schafer JC, Baetz NW, Lapierre LA, McRae RE, Roland JT, Goldenring JR 2014 Rab11-FIP2 interaction with MYO5B regulates movement of Rab11a-containing recycling vesicles. Traffic 15:292–308. doi:10.1111/tra.12146. 24372966 PMC4081500

[20] Avilov SV, Moisy D, Munier S, Schraidt O, Naffakh N, Cusack S 2012 Replication-competent influenza A virus that encodes a split-green fluorescent protein-tagged PB2 polymerase subunit allows live-cell imaging of the virus life cycle. J Virol 86:1433–1448. doi:10.1128/JVI.05820-11. 22114331 PMC3264389

[21] Arcangeletti MC, De Conto F, Ferraglia F, Pinardi F, Gatti R, Orlandini G, Covan S, Motta F, Rodighiero I, Dettori G, Chezzi C 2008 Host-cell-dependent role of actin cytoskeleton during the replication of a human strain of influenza A virus. Arch Virol 153:1209–1221. doi:10.1007/s00705-008-0103-0. 18488136

[22] Kumakura M, Kawaguchi A, Nagata K 2015 Actin-myosin network is required for proper assembly of influenza virus particles. Virology 476:141–150. doi:10.1016/j.virol.2014.12.016. 25543965

[23] Roberts PC, Compans RW 1998 Host cell dependence of viral morphology. Proc Natl Acad Sci U S A 95:5746–5751. doi:10.1073/pnas.95.10.5746. 9576955 PMC20450

[24] Simpson-Holley M, Ellis D, Fisher D, Elton D, McCauley J, Digard P 2002 A functional link between the actin cytoskeleton and lipid rafts during budding of filamentous influenza virions. Virology 301:212–225. doi:10.1006/viro.2002.1595. 12359424

[25] Momose F, Kikuchi Y, Komase K, Morikawa Y 2007 Visualization of microtubule-mediated transport of influenza viral progeny ribonucleoprotein. Microbes Infect 9:1422–1433. doi:10.1016/j.micinf.2007.07.007. 17905627

[26] Avilov SV, Moisy D, Naffakh N, Cusack S 2012 Influenza A virus progeny vRNP trafficking in live infected cells studied with the virus-encoded fluorescently tagged PB2 protein. Vaccine 30:7411–7417. doi:10.1016/j.vaccine.2012.09.077. 23063830

[27] Kawaguchi A, Asaka MN, Matsumoto K, Nagata K 2015 Centrosome maturation requires YB-1 to regulate dynamic instability of microtubules for nucleus reassembly. Sci Rep 5:8768. doi:10.1038/srep08768. 25740062 PMC4350100

[28] Kawaguchi A, Matsumoto K, Nagata K 2012 YB-1 functions as a porter to lead influenza virus ribonucleoprotein complexes to microtubules. J Virol 86:11086–11095. doi:10.1128/JVI.00453-12. 22855482 PMC3457152

[29] Bruce EA, Digard P, Stuart AD 2010 The Rab11 pathway is required for influenza A virus budding and filament formation. J Virol 84:5848–5859. doi:10.1128/JVI.00307-10. 20357086 PMC2876627

[30] Hales CM, Griner R, Hobdy-Henderson KC, Dorn MC, Hardy D, Kumar R, Navarre J, Chan EK, Lapierre LA, Goldenring JR 2001 Identification and characterization of a family of Rab11-interacting proteins. J Biol Chem 276:39067–39075. doi:10.1074/jbc.M104831200. 11495908

[31] Casanova JE, Wang X, Kumar R, Bhartur SG, Navarre J, Woodrum JE, Altschuler Y, Ray GS, Goldenring JR 1999 Association of Rab25 and Rab11a with the apical recycling system of polarized Madin-Darby canine kidney cells. Mol Biol Cell 10:47–61. doi:10.1091/mbc.10.1.47. 9880326 PMC25153

[32] Widdicombe JH, Sachs LA, Finkbeiner WE 2003 Effects of growth surface on differentiation of cultures of human tracheal epithelium. In Vitro Cell Dev Biol Anim 39:51–55. doi:10.1290/1543-706X(2003)039<0051:EOGSOD>2.0.CO;2. 12892527

[33] Piperno G, LeDizet M, Chang XJ 1987 Microtubules containing acetylated alpha-tubulin in mammalian cells in culture. J Cell Biol 104:289–302. doi:10.1083/jcb.104.2.289. 2879846 PMC2114420

[34] Quinones GB, Danowski BA, Devaraj A, Singh V, Ligon LA 2011 The posttranslational modification of tubulin undergoes a switch from detyrosination to acetylation as epithelial cells become polarized. Mol Biol Cell 22:1045–1057. doi:10.1091/mbc.E10-06-0519. 21307336 PMC3069008

[35] Husain M, Harrod KS 2011 Enhanced acetylation of alpha-tubulin in influenza A virus infected epithelial cells. FEBS Lett 585:128–132. doi:10.1016/j.febslet.2010.11.023. 21094644

[36] Wloga D, Gaertig J 2010 Post-translational modifications of microtubules. J Cell Sci 123:3447–3455. doi:10.1242/jcs.063727. 20930140 PMC2951466

[37] Hutchinson EC, von Kirchbach JC, Gog JR, Digard P 2010 Genome packaging in influenza A virus. The J Gen Virol 91:313–328. doi:10.1099/vir.0.017608-0. 19955561

[38] Shaw ML, Stone KL, Colangelo CM, Gulcicek EE, Palese P 2008 Cellular proteins in influenza virus particles. PLoS Pathog 4:e1000085. doi:10.1371/journal.ppat.1000085. 18535660 PMC2390764

[39] Valm AM, Cohen S, Legant WR, Melunis J, Hershberg U, Wait E, Cohen AR, Davidson MW, Betzig E, Lippincott-Schwartz J 2017 Applying systems-level spectral imaging and analysis to reveal the organelle interactome. Nature 546:162–167. doi:10.1038/nature22369. 28538724 PMC5536967

[40] Myerburg MM, Harvey PR, Heidrich EM, Pilewski JM, Butterworth MB 2010 Acute regulation of the epithelial sodium channel in airway epithelia by proteases and trafficking. Am J Respir Cell Mol Biol 43:712–719. doi:10.1165/rcmb.2009-0348OC. 20097829 PMC2993091

[41] Lakdawala SS, Lamirande EW, Suguitan AL Jr, Wang W, Santos CP, Vogel L, Matsuoka Y, Lindsley WG, Jin H, Subbarao K 2011 Eurasian-origin gene segments contribute to the transmissibility, aerosol release, and morphology of the 2009 pandemic H1N1 influenza virus. PLoS Pathog 7:e1002443. doi:10.1371/journal.ppat.1002443. 22241979 PMC3248560

[42] Chen Z, Wang W, Zhou H, Suguitan AL Jr, Shambaugh C, Kim L, Zhao J, Kemble G, Jin H 2010 Generation of live attenuated novel influenza virus A/California/7/09 (H1N1) vaccines with high yield in embryonated chicken eggs. J Virol 84:44–51. doi:10.1128/JVI.02106-09. 19864389 PMC2798434

[43] Bjaelde RG, Arnadottir SS, Leipziger J, Praetorius HA 2011 Agonists that increase [Ca^2+^](i) halt the movement of acidic cytoplasmic vesicles in MDCK cells. J Membr Biol 244:43–53. doi:10.1007/s00232-011-9396-0. 21989951

[44] Breitfeld PP, McKinnon WC, Mostov KE 1990 Effect of nocodazole on vesicular traffic to the apical and basolateral surfaces of polarized MDCK cells. J Cell Biol 111:2365–2373. doi:10.1083/jcb.111.6.2365. 2277063 PMC2116403

[45] Hunziker W, Male P, Mellman I 1990 Differential microtubule requirements for transcytosis in MDCK cells. EMBO J 9:3515–3525. 2170116 10.1002/j.1460-2075.1990.tb07560.x PMC552100

[46] Lapierre LA, Dorn MC, Zimmerman CF, Navarre J, Burnette JO, Goldenring JR 2003 Rab11b resides in a vesicular compartment distinct from Rab11a in parietal cells and other epithelial cells. Exp Cell Res 290:322–331. doi:10.1016/S0014-4827(03)00340-9. 14567990

[47] Ojakian GK, Schwimmer R 1992 Antimicrotubule drugs inhibit the polarized insertion of an intracellular glycoprotein pool into the apical membrane of Madin-Darby canine kidney (MDCK) cells. J Cell Sci 103(Pt 3):677–687. 1478964 10.1242/jcs.103.3.677

[48] De Conto F, Di Lonardo E, Arcangeletti MC, Chezzi C, Medici MC, Calderaro A 2012 Highly dynamic microtubules improve the effectiveness of early stages of human influenza A/NWS/33 virus infection in LLC-MK2 cells. PLoS One 7:e41207. doi:10.1371/journal.pone.0041207. 22911759 PMC3401105

[49] Roberts KL, Manicassamy B, Lamb RA 2015 Influenza A virus uses intercellular connections to spread to neighboring cells. J Virol 89:1537–1549. doi:10.1128/JVI.03306-14. 25428869 PMC4300760

[50] Akopova I, Tatur S, Grygorczyk M, Luchowski R, Gryczynski I, Gryczynski Z, Borejdo J, Grygorczyk R 2012 Imaging exocytosis of ATP-containing vesicles with TIRF microscopy in lung epithelial A549 cells. Purinergic Signal 8:59–70. doi:10.1007/s11302-011-9259-2. 21881960 PMC3286538

[51] Bose S, Malur A, Banerjee AK 2001 Polarity of human parainfluenza virus type 3 infection in polarized human lung epithelial A549 cells: role of microfilament and microtubule. J Virol 75:1984–1989. doi:10.1128/JVI.75.4.1984-1989.2001. 11160698 PMC115145

[52] dos Santos T, Varela J, Lynch I, Salvati A, Dawson KA 2011 Effects of transport inhibitors on the cellular uptake of carboxylated polystyrene nanoparticles in different cell lines. PLoS One 6:e24438. doi:10.1371/journal.pone.0024438. 21949717 PMC3176276

[53] Horibe S, Matsuda A, Tanahashi T, Inoue J, Kawauchi S, Mizuno S, Ueno M, Takahashi K, Maeda Y, Maegouchi T, Murakami Y, Yumoto R, Nagai J, Takano M 2015 Cisplatin resistance in human lung cancer cells is linked with dysregulation of cell cycle associated proteins. Life Sci 124:31–40. doi:10.1016/j.lfs.2015.01.011. 25625243

[54] Schwingshackl A, Roan E, Teng B, Waters CM 2015 TREK-1 regulates cytokine secretion from cultured human alveolar epithelial cells independently of cytoskeletal rearrangements. PLoS One 10:e0126781. doi:10.1371/journal.pone.0126781. 26001192 PMC4441361

[55] Reed LJ, Muench H 1938 A simple method of estimating fifty percent endpoints. Am J Epidemiol 27:493–497. doi:10.1093/oxfordjournals.aje.a118408.

